# Antimicrobial Activity of Polycaprolactone Nanofiber Coated with Lavender and Neem Oil Nanoemulsions against Airborne Bacteria

**DOI:** 10.3390/membranes14020036

**Published:** 2024-01-29

**Authors:** Md Mahfuzur Rahman, Hari Kotturi, Sadegh Nikfarjam, Kanika Bhargava, Nagib Ahsan, Morshed Khandaker

**Affiliations:** 1 Department of Human Environmental Sciences, University of Central Oklahoma, Edmond, OK 73034, USA; mrahman24@uco.edu (M.M.R.); kbhargava@uco.edu (K.B.); 2 Department of Biology, University of Central Oklahoma, Edmond, OK 73034, USA; snikfarjam@uco.edu; 3 Department of Chemistry and Biochemistry, The University of Oklahoma, Norman, OK 73019, USA; nahsan@ou.edu; 4 Mass Spectrometry, Proteomics and Metabolomics Core Facility, Stephenson Life Sciences Research Center, University of Oklahoma, Norman, OK 73019, USA; 5 Nanobiology Laboratory, School of Engineering, University of Central Oklahoma, Edmond, OK 73034, USA

**Keywords:** neem oil, lavender oil, *E. coli*, *B. subtilis*, *S. aureus*, nanoemulsion, antimicrobial activity, sonication

## Abstract

The development of efficient, eco-friendly antimicrobial agents for air purification and disinfection addresses public health issues connected to preventing airborne pathogens. Herein, the antimicrobial activity of a nanoemulsion (control, 5%, 10%, and 15%) containing neem and lavender oils with polycaprolactone (PCL) was investigated against airborne bacteria, including *Escherichia coli*, *Bacillus subtilis*, and *Staphylococcus aureus*. Various parameters such as the physicochemical properties of the nanoemulsion, pH, droplet size, the polydispersity index (PDI), the minimum inhibitory concentration (MIC), the minimum bacterial concentration (MBC), and the color measurement of the emulsion have been evaluated and optimized. Our results showed that the antimicrobial activity of PCL combined with neem and lavender oil was found to be the highest MIC and MBC against all tested bacteria. The droplet sizes for lavender oil are 21.86–115.15 nm, the droplet sizes for neem oil are 23.92–119.15 nm, and their combination is 25.97–50.22 nm. The range of pH and viscosity of nanoemulsions of various concentrations was found to be 5.8 to 6.6 pH and 0.372 to 2.101 cP. This study highlights the potential of nanotechnology in harnessing the antimicrobial properties of natural essential oils, paving the way for innovative and sustainable solutions in the fight against bacterial contamination.

## 1. Introduction

Numerous scientific studies have demonstrated the antimicrobial and anti-inflammatory properties of natural oils, including lavender and neem [1,2,3,4]. Neem oil, derived from neem tree seeds, is a natural insecticide and organic fungicide with antimicrobial properties, effectively repelling pests and preventing fungal diseases in plants [1]. Neem oil is a key ingredient in cosmetic and skincare products due to its ability to soothe skin conditions, like acne and eczema. Furthermore, its high vitamin E content aids in promoting hair growth and scalp health. Neem oil is employed for traditional medicinal purposes, treating ailments such as fever and digestive issues. Its versatile uses make it a valuable addition to both natural medicine and sustainable agriculture [2,3]. Lavender oil, extracted from the lavender plant, has therapeutic properties and is used in aromatherapy, personal care, and natural cleaning products. It promotes relaxation, reduces stress, and improves sleep quality. It also has antiseptic and anti-inflammatory properties, making it effective in treating minor burns, insect bites, and skin irritations [4]. Lavender oil’s pleasant scent and antimicrobial properties make it a valuable addition to holistic health. Nanoemulsions are colloidal systems that are made by shearing two non-mixing liquids with the help of external energy and stabilizing with surfactants. Lavender oil, rich in linalool and linalyl acetate, adds broad-spectrum antimicrobial action. It is widely classified as oil-in-water (o/w) or water-in-oil (w/o) systems. Both systems are kinetically stabilized and exhibit droplet sizes in the range of 20–1000 nm [2,5]. The optical appearance of these systems was reported to range from turbid to transparent and was dependent on droplet size [6]. The application of face masks made from polycaprolactone nanofiber coated with lavender and neem oil nanoemulsions presents an innovative approach to combating airborne bacteria. For the nanofiber mask, polycaprolactone, a biodegradable polyester, is the foundation material. After that, nanoemulsions of neem and lavender oil, which are both well-known for having inherent antibacterial qualities, are applied to it. While neem oil is known for its broad-spectrum antibacterial properties, lavender oil has a calming scent and is known to relieve tension [7]. By performing this, the mask should be better able to filter and destroy airborne germs, acting as a natural and efficient barrier against microbial contamination of the air. The research provides a possible development in protective face masks with natural antibacterial agents by evaluating the masks’ breathability, filtration efficiency, antibacterial qualities, and user comfort.

Airborne bacteria such as *Escherichia coli*, *Bacillus subtilis*, and *Staphylococcus aureus* showed unique characteristics. *E. coli*, a Gram-negative bacterium, is used as an indicator organism for fecal contamination and presents significant health risks. *Bacillus subtilis*, a Gram-positive spore-forming bacterium, can persist in the environment and impact indoor air quality [8]. *Staphylococcus aureus*, another Gram-positive bacterium, is a common human pathogen causing skin infections and more severe diseases. The antimicrobial activity of nanoemulsions containing neem and lavender oils is noteworthy in combating airborne bacteria. Neem oil, with its potent antibacterial and antifungal properties, works synergistically with lavender oil’s antimicrobial and soothing qualities [9]. The nanoemulsion formulation enhances their effectiveness by reducing droplet size, increasing surface area, and promoting better dispersion. A nanoemulsion containing neem and lavender oils represents a sophisticated blend of natural ingredients in a highly advanced formulation. Neem oil, derived from the neem tree, is renowned for its medicinal properties, including anti-inflammatory, antibacterial, and antifungal activities.

The polydispersity index (PDI) and droplet size are crucial variables. The PDI is used to characterize the extent of non-uniformity in the size distribution of particles present in a sample, such as a nanoemulsion or colloidal suspension. An indicator of the distribution spread of the particle sizes is provided by the PDI, a dimensionless quantity. The most common method to determine the PDI is through Dynamic Light Scattering (DLS). This technique measures the time-dependent fluctuations in the scattering of light due to the Brownian motion of the particles. A low PDI suggests droplet size homogeneity, which is essential for the stability and consistency of the nanoemulsion. The droplet size impacts the surface area and rate of release of the active components. The therapeutic characteristics of neem and lavender oil may be effectively given through a well-formulated nanoemulsion with small, homogeneous droplet sizes (low PDI). This makes the oil appropriate for a wide range of uses in skincare, health, and wellness products [1,2].

Polycaprolactone (PCL) is a biodegradable polyester commonly used in various industries. It has a low melting point, high flexibility, and good biocompatibility, making it suitable for applications, such as drug delivery, tissue engineering, 3D printing, and it is also a scaffold material for regenerative medicine due to its slow biodegradation rate. The antimicrobial activity of PCL-incorporated nanoemulsions containing neem and lavender oil presents a powerful tool in the fight against pathogenic microorganisms [10]. Neem oil is well-known for its broad-spectrum antimicrobial properties, capable of inhibiting the growth of bacteria, fungi, and even certain viruses [2,3]. Lavender oil complements this effect with its natural antibacterial and antifungal characteristics. When these oils are encased in PCL nanoparticles, they can be delivered to specific areas, and the antimicrobial agents can be released slowly and steadily. This technology holds immense potential in medical devices, wound dressings, and pharmaceuticals, where preventing infections is critical [11]. Additionally, it can find applications in cosmetic and personal care products, enhancing their safety and effectiveness by reducing the risk of microbial contamination. PCL-incorporated nanoemulsions offer a versatile and environmentally friendly solution for combating harmful microorganisms across various industries [12]. In this study, we prepared neem and lavender oil nanoemulsions and optimized their physicochemical characteristics, droplet size, polydispersity index (PDI), concentration, and combination of the nanoemulsions for MIC and MBC against common airborne bacteria.

## 2. Materials and Methods

### 2.1. Materials and Test Organisms

Commercially available neem and lavender oils were purchased from a local market in Edmond, Oklahoma, and stored at room temperature. Sterile deionized water and Tween 80 (polysorbate 80, Sigma Aldrich, St. Louis, MI, USA) were used for all experiments as solvents. ATCC-confirmed strains of *Escherichia coli*, *Bacillus subtilis*, and *Staphylococcus aureus* were provided by the Department of Biology, University of Central Oklahoma, Edmond, Oklahoma. All the bacterial strains were cultured in nutrient broth and plated on nutrient agar media.

### 2.2. Production of Polycaprolactone Nanofiber

In our study, we produced polycaprolactone (PCL) nanofiber membranes using ENF Product, LLC (Edmond, OK, USA, www.enfproducts.com, accessed on 20 October 2023) electrospinning apparatus. This process involved dissolving PCL in acetone to create a solution, which was then fed through a single-axis, one-inch discharge metallic needle (Model # BX 25). The electrospinning machine included a drum collector, operated by speed-controlled direct current (DC) motors. A high voltage of 9 kV, generated by a high precision and high voltage power supply AC-DC conversion MAX output −20 KV 0.5 mA (Analog Technologies, Inc., San Jose, CA, USA), was applied to the syringe needle, creating an electrically charged jet in the PCL solution. This jet was directed toward the drum collector, located approximately 5 cm away from the needle at room temperature and relative humidity of 30–40%, to form a stream of synthetic polymer fibers [13]. We fine-tuned several parameters, including the rotation speed of the drum, the needle–drum distance, and the fiber deposition rate, to optimize fiber mat formation. The solution feeding rate was set to 0.025 mL/minute, and the fibers were collected on a drum with a radius of 40 mm.

### 2.3. Preparation of Nanoemulsion

PCL nanofiber cloth was prepared at the nanobiology lab, Department of Engineering, University of Central Oklahoma in Edmond, Edmond, OK, USA. The detailed method of the PCL manufacturing process and characterization is given by Khandaker et al. [13]. In short, PCL was dissolved in acetone by an ultrasonic mixer. The resulting solution was electrospun using a custom-made electrospun nanofiber machine housed in the lab. The PCL fiber cloth is deposited on a drum collector and cut into 1 cm disks using a circular punch for analysis. This study prepared three groups of nanoemulsion immobilized PCL for physical, chemical, and antibacterial activities and compared those values with PCL. The prepared nanoemulsion groups are neem nanoemulsion, lavender nanoemulsion, and a combined nanoemulsion containing both neem and lavender oils. Each neem and lavender nanoemulsion was prepared at 5%, 10%, and 15% concentrations, where Tween 80 and deionized water were used as solvents. A magnetic stir plate was mixed with each group of nanoemulsion solutions for 30 min at 700 rpm. To prepare neem and lavender oil nanoemulsion, each oil was mixed at a ratio of 1:1, and then the mixer was used to prepare 5%, 10%, and 15% concentrations of neem and lavender oil nanoemulsion solutions. An ultrasonicator (Qsonica, Model # CL334, Newtown, CT, USA) was used to produce nanoemulsion, according to the protocol discussed in detail in Paudel et al. [14]. The used wattage was between 50 and 70. The duration of the emulsion pulse was 5 s at the start and 3 s at the end. The probe’s dimensions are 0.5 inches in diameter, 1.905 cm in depth, and 60 in terms of amplitude. To prevent heating, the experiment was conducted in an ice bath. Each sample was ultrasonically treated for 20 min. The PCL samples were soaked in nanoemulsion at different concentrations. Control samples are the PCL disk without the soaking of neem, lavender, or neem–lavender nanoemulsion solutions.

### 2.4. Scanning Electron Microscopy (SEM) Characterization

PCL and PCL nanoemulsion scanning electron micrographs were produced using a Zeiss Neon 40 EsB apparatus. At 50,000×, the samples were directly scanned at 5 kV of acceleration. A Thermo Quattro S-Field Emission Environmental Scanning Electron Microscope, operating at 5000× without staining, and a 20 kV accelerating voltage were used to examine the interaction between PCL and the PCL nanoemulsion nanofiber. 

### 2.5. Measurement of Nanoemulsion’s Physiochemical Properties

The pH of the nanoemulsion was determined according to the earlier published protocol [13]. To measure the viscosity of the nanoemulsion, we used a Kinexus rheometer (KNX0226) with a 40 mm rheometer plate at 25 °C and a measured gap of 1 mm to figure out the shear viscosities of the nanoemulsion samples. Before measurement, the NE samples were allowed to acclimate on the rheometer plate for 5 min. To describe the rheological characteristics of the NE samples, the viscosity values at shear rates ranging from 0.1 to 200/s were measured [15]. According to Kotturi et al., 2022, experiments on water absorption were carried out [16]. Three 1-inch-diameter PCL pieces were cut, and we immersed each one in nanoemulsion for five minutes. Each item took thirty minutes to air dry. A precise scale was used to measure each sample’s weight both before and after it was soaked. The water absorptions were calculated by Equation (1):

(1) $$Water\;absorption\;rate\;\left(\%\right)=\frac{W_{a}-W_{b}}{W_{b}}\times 100\%$$

where *W_a_* is the weight of the material after absorption and *W_b_* is the weight of the material before absorption.

Nanoemulsion color measurement by a colorimeter (Shenzhen Linshang Technology Co., Ltd., Model: LS172, Baoan, Shenzhen, China) can be quantified using the CIE Lab* color space, a standard method in color science [17]. The Lab* color space describes color in terms of three coordinates: L* for lightness, a* for red-green, and b* for yellow-blue. The formula for color difference (Δ*E*) between two samples in the CIE Lab* space was calculated using Equation (2).

(2) $$\Delta E=\sqrt{\Delta {L^{*}}^{2}+\Delta {a^{*}}^{2}+\Delta {b^{*}}^{2}}$$

where ΔL*, Δa*, and Δb* represent the differences in L*, a*, and b* values between the nanoemulsion and a reference standard. This equation quantifies color changes in nanoemulsions, aiding in quality control and product development in various industries.

A disk diffusion assay was performed using 1 cm diameter sterile PCL nanofiber disks coated with 5%, 10%, and 15% nanoemulsions. Briefly, Muller–Hinton agar plates were coated with 0.5 McFarland overnight bacterial cultures of *E. coli*, *S. aureus*, and *B. subtilis.* The PCL nanoemulsion disk was placed in the center of the plate. The plates were incubated at 37 °C for 24, 48, and 72 h. The zones of inhibition were recorded using a caliper, and 5 values were taken for each zone and recorded. The assay was performed in triplicate with PCL disk controls without nanoemulsions [14].

### 2.6. Emulsion Characterization

The experimental method of emulsion characterization using the pUNk Dynamic Light Scattering (DLS) system involves preparing a stable emulsion, setting up the pUNk DLS system, and then loading the emulsion into the sample chamber. The particle size distribution of the sample is determined by analyzing the scattered light intensity variations that are recorded when the device lights it with a laser [18]. DLS provides data on the size distribution of the particles. The PDI is calculated based on the width of this size distribution. It is calculated using the formula PDI = (mean diameter of particle size)^2^/(standard deviation of the size distribution of the particles)^2^. Through the use of pUNk DLS software, Unchained Labs, Pleasanton, CA, USA, PDI data were calculated to provide information on the emulsion’s stability, homogeneity, and particle size. 

### 2.7. Antimicrobial Assay

In this study, we evaluated the antimicrobial efficacy of neem and lavender oil nanoemulsions using the broth dilution method, as outlined in the Clinical and Laboratory Standards Institute’s M07-A8 protocol. Our experimental setup consisted of Muller–Hinton broth, inoculated with a 0.5 McFarland Standard suspension of *Escherichia coli*, *Staphylococcus aureus*, and *Bacillus subtilis*. For each microbial culture, sterile polycaprolactone (PCL) nanofiber disks, each 1 cm in diameter, were treated with nanoemulsions containing 5%, 10%, or 15% concentrations of either neem oil, lavender oil, or a combination of both. These treated disks were then introduced to the respective bacterial cultures and incubated at 37 °C for 24 h [19]. Post-incubation, we assessed the cultures for microbial growth to determine the antimicrobial activity of the nanoemulsions. Control groups for this experiment included nanoemulsions devoid of neem or lavender oils. We conducted each assay in triplicate to ensure the reliability of our data and meticulously recorded all observations and measurements. The bacterial inhibition equation was calculated by Equation (3).

(3) $$\%\;\mathrm{of}\;\mathrm{inhibition}=\frac{A-B}{A}\times 100\%$$

where *A* is the bacterial growth in the control (without the antibacterial agent) and *B* is the bacterial growth in the presence of the antibacterial agent.

### 2.8. Minimum Inhibitory Concentration (MIC) and Minimum Bactericidal Concentration (MBC) Determination

The antimicrobial efficacy of nanoemulsion was evaluated using the standard broth dilution method (CLSI M07-A8). Serial two-fold dilutions were conducted to determine the minimum inhibitory concentration (MIC) in nutrient broth, with visual turbidity being noted before and after incubation (24 h at 37 °C) to confirm the MIC value [20]. The bactericidal effect of our nanoemulsion was confirmed using an MBC assay using published protocols [20]. Briefly, 5 mls Muller–Hinton broth containing 0.5 McFarland Standard of *E. coli*, *S. aureus*, and *B. subtilis* were used for all our experiments. Sterile PCL nanofiber disks 1 cm in diameter treated with 5%, 10%, and 15% nanoemulsions of either neem, or, lavender, or their combination were added to the bacterial culture and incubated for 24 h at 37 °C. After incubation, MBC was confirmed by streaking a loopful of the incubated onto a Muller–Hinton agar plate. The plates were incubated for an additional 24 h at 37 °C and examined for the growth of bacteria. This assay was performed in triplicates with proper controls. 

### 2.9. Statistical Analyses

Using IBM SPSS Statistics (version 22, SPSS Inc., Chicago, IL, USA), the data were assessed. The findings were presented as the SE of the mean across three replicates. A one-way analysis of variance with a 95% confidence level was used to evaluate statistically significant differences between the means.

## 3. Results and Discussion

### 3.1. Physicochemical Characterization of Nanoemulsion

**Figure 1:**
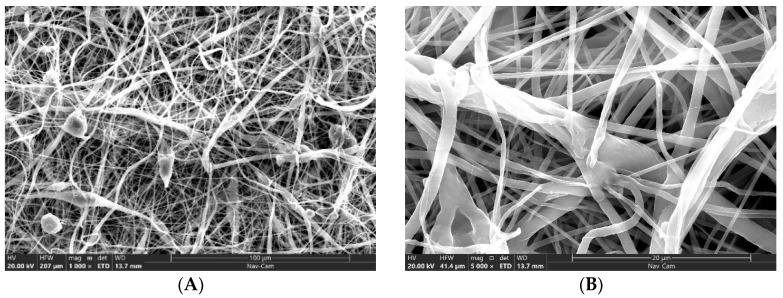

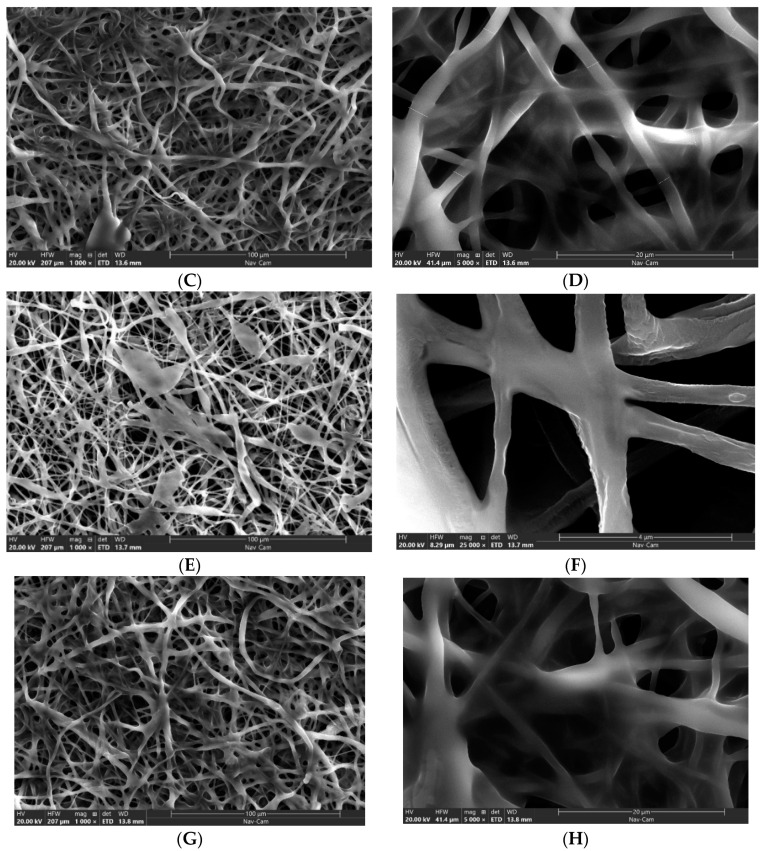
SEM images ((**A**,**B**)–only PCL), ((**C**,**D**)–PCL + neem oil nanoemulsion), ((**E**,**F**)–PCL + lavender oil nanoemulsion), and ((**G**,**H**)–PCL + lavender + neem oil). Left column pictures were captured at 1000× maginficaiton, where right column pictures were captured at 5000×. The 5000× images were captured from an arbitrary location. There is clear difference of nanofiber diameter among the samples.was observed from the images.

Scanning electron microscopy (SEM) images of polycaprolactone (PCL) nanofiber cloth infused with nanoemulsions of neem and lavender oil are depicted in Figure 1. Initially, the average diameter of PCL nanofibers is approximately 50 ± 12 nm. However, following the absorption of the nanoemulsion, the average diameter increases to about 100 ± 32 nm. At a magnification of 50,000×, the fusion of the fiber and nanoemulsion is visible. Images A and B show the PCL nanofibers alone, while images C and D display PCL nanofibers after the absorption of the neem oil nanoemulsion. This study focuses on the SEM imagery of neem oil nanoemulsions encapsulated within PCL. Neem oil is characterized by its high viscosity, resulting in a viscous and sticky appearance in the images. The SEM images also highlight the uniformity and size of the nanoemulsion droplets, reflecting the effectiveness of the encapsulation process. The nanofiber size of PCL changes after immersion in the neem oil nanoemulsion. In contrast, images E and F illustrate PCL nanofibers with lavender oil nanoemulsion, which are less viscous than neem oil. Finally, images E and F also present the PCL nanofibers with a combined nanoemulsion of lavender and neem oil in this study.

The viscosity of neem, lavender, and neem–lavender nanoemulsion formulations were examined to evaluate the soaking capabilities of the nanoemulsion with the PCL nanofiber matrix. Our results indicate that the viscosities of 5%, 10%, and 15% neem oil were 2.101, 1.807, and 1.440 cP, respectively. A previous study also reported that the viscosity of neem oil was 2.11 cP, which is an almost similar value [2]. Because the nanoemulsion was uniform and had low viscosity during formulation, nanodroplets increased the interfacial area. This increased interfacial area resulted due to non-solvant characteristic of oil in water, which improved the mixer ability to move [21]. On the other hand, the viscosities of 5%, 10%, and 15% lavender oil were found to be 2.047, 1.050, and 0.548 cP, respectively (Table 1). A previous study also reported that the viscosity of lavender oil was 5.67 cP, which is two times higher than the study value [4]. Similarly, the viscosity of neem and lavender oil nanoemulsions with 5%, 10%, and 15% was found to be 1.840, 0.726, and 0.372 cP, respectively. Neem and lavender oils’ viscosity affects their flow, dispersion, and application qualities, which in turn affect how effective they are for a variety of tasks, from cosmetics to agriculture [22]. The pH range of neem, lavender neem, and lavender oil was found to range from 5.9 to 6.5, 5.8 to 6.3, and 6.2 to 6.6 in this study (Table 1). To maintain the stability of the emulsion system, pH is crucial. Neem nanoemulsion pH values ranged from 3.53 to 4.51 [22], whereas lavender oil nanoemulsion pH values ranged from 6.3 to 6.65 [4]. There was no correlation between the pH alteration and the amount of plant oils in nanoemulsions.

Polycaprolactone (PCL) is a biodegradable polyester with a low melting point of around 60 °C and is often used in medical applications for the decontamination of face musk. The microscopic droplets in the emulsion may be absorbed into the PCL matrix when PCL and nanoemulsions are mixed [16]. In this research, the initial weight of the PCL clothing rises after soaking and increases four times in the final PCL weights.

Water absorption properties of polycaprolactone (PCL) nanofibers and emulsions are detailed in Table 1. These nanofibers demonstrate a notable capacity for water absorption and retention, attributed to their high surface area-to-volume ratio. This characteristic renders them particularly advantageous for various applications, including filtration systems, sensor technology, and the manufacturing industry. In the realm of experimental observations, the research highlights a significant variance in water absorption rates depending on the composition of the nanofibers. Specifically, the incorporation of 15% lavender oil in the nanofibers yielded the highest water absorption rate, recorded at 31.45%. Conversely, the lowest absorption rate observed in this study was 14.14%. These findings underscore the potential of modifying PCL nanofibers with different additives to tailor their absorption properties for specific industrial applications.

### 3.2. NanoEmulsion Droplet Size and Polydispersity Index

In order for nanoemulsions to be effective and functional, the droplet size and polydispersity index (PDI) are crucial. Particularly important in the food, pharmaceutical, and cosmetic industries, nanoemulsion droplet size greatly affects properties, including stability, bioavailability, and absorption rate. The performance and distribution of active substances are improved by smaller droplets since they have a greater surface area. Analogously significant is the PDI, which shows homogeneity of droplet sizes. A lower PDI indicates a distribution that is more uniform, which is necessary for consistent product quality and performance. The droplet size and polydispersity index of nanoemulsions at 4 °C, 25 °C, and 37 °C are presented in Table 2. In this study, the highest droplet size diameter of the control (5%) was 105.52 nm at 37 °C, whereas the lowest droplet size diameter of the control (15%) was 9.04 nm at 4 °C. A previous study also reported that the droplet size of lavender oil was found to be 104.55 nm [4], whereas our study value is 135.75 nm. Similarly, the highest PDI of the control (5%) was 16.38% at 37 °C, whereas the lowest PDI of the control (10%) was 11.69% at 4 °C. Moreover, the highest droplet size diameter of the neem oil emulsion (5%) was 14.81 nm at 37 °C, whereas the lowest droplet size diameter of the neem oil emulsion (15%) was 10.04 nm at 4 °C. Another study reported that the polydispersity index (PDI) of neem oil was found to be 42.8% [23], whereas our experimental result in this study is 13.37%, which is more than three times smaller than the reported values.

The temperature has a major effect on the polydispersity index (PDI) and droplet size in nanoemulsions. The kinetic energy of the droplets increases with temperature, which may cause changes in droplet size because of more frequent collisions and coalescence. Larger droplet sizes and a greater PDI may arise from this, suggesting a wider size dispersion [5,14]. On the other hand, decreased kinetic energy at lower temperatures may preserve or even shrink droplet size, improving stability and lowering the PDI. This study value also represents the same data: temperature increases, droplet size increases, and the PDI value also increases (Table 2). For stability, increased bioavailability, and efficient administration of therapeutic effects in a variety of health and skincare applications, ideal droplet size and a low PDI are required in essential oil nanoemulsions.

### 3.3. Nanoemulsion Color Measurement

Color is a critical indicator of stability and quality in nanoemulsions, as color changes can signify degradation, phase separation, or other undesirable alterations in the formulation. Nanoemulsions’ color can be influenced by emulsifying agents, oil phase composition, droplet size, and bioactive compounds. Spectrophotometric analysis can quantify hue, chroma, and lightness, which are crucial for food, pharmaceutical, and cosmetic applications, as color affects product quality and consumer acceptance [24]. The color properties (L*, *a*, *b*, Δ*E*, *C*, and *H*) of the nanoemulsion of control, neem, lavender oil, and their mixture are presented in Table 3. The control values of the emulsion ranges L* (42.36–44.14), a* (−5.89–8.12), and b* (9.99–17.15) were measured. Similarly, the neem oil L* value was measured in the range of 78.22–79.60, lavender oil was measured in the range of 74.39–82.32, and neem + lavender oil was measured in the range of 77.97–81.13. Neem oil’s color measurements in a related investigation revealed L* 46.1, a* 12.2, and b* 35.7 [25]. A considerable yellow parameter (+b*) can be seen in this work when the given measure conditions are used, in contrast to a weak red value (+a*), which coupled with the rather brilliant L* value accounts for the light reddish-brown hue of the neem oil.

### 3.4. Evaluation of Nanoemulsion’s Antimicrobial Activity

The pH of our nanoemulsion formulations of neem, lavender, and mixed oil at 5%, 10%, and 15% were presented in Table 1. This experiment used an ultrasonic emulsion technique to create the nanoemulsion. Ultrasonication is a high-energy technique for creating nanoemulsions, and the emulsions it produces have smaller, more evenly distributed water droplets, greater emulsion stability, and smaller droplet sizes [26]. 

**Figure 2:**
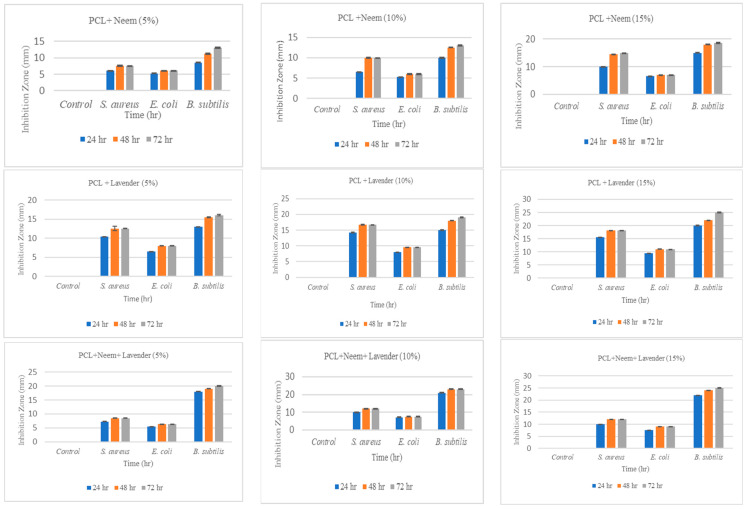
Effects of nanoemulsion-treated PCL on the survival of *S. aureus*, *E. coli,* and *B. subtilis* at 24, 48, and 72 h using a disk diffusion assay.

**Figure 3:**
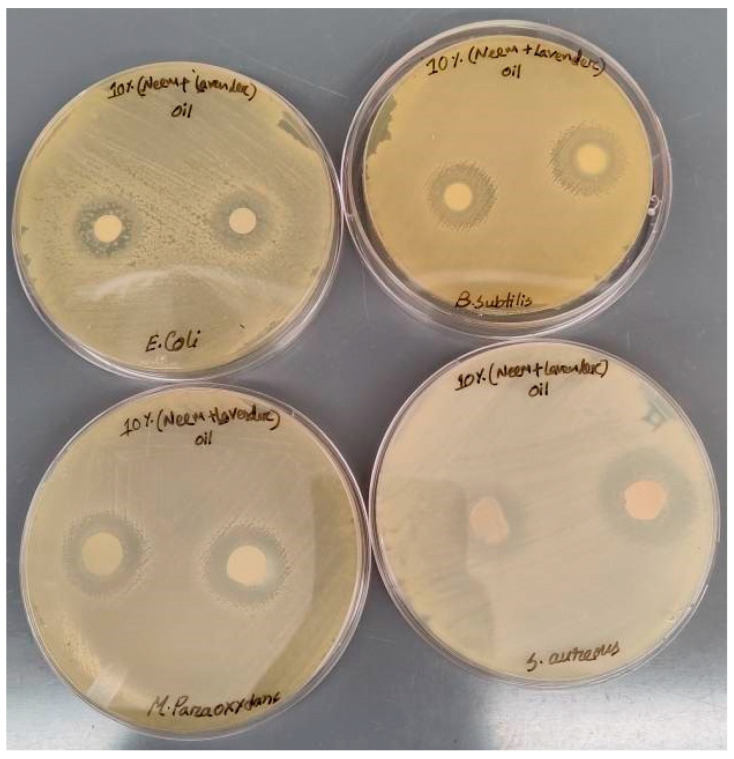
A disk diffusion assay image used to calculate the zone of inhibition of study samples.

A disk diffusion assay was performed as described above, and the plates were incubated for 24, 48, and 72 h with nanoemulsion controls. The diameter of zones of inhibition was measured at five diagonal points using a caliper. The antimicrobial activity of PCL + neem oil, PCL + lavender oil, and PCL + neem oil + lavender oil at 5%, 10%, and 15% concentrations against *E. coli*, *S. aureus*, and *B. subtilis* is presented in Figure 2. After 24 h of incubation, the diameter of the zone of inhibition (PCL + 5% neem oil) was 8.5 mm for *B. subtilis*. Figure 2 was created based on a total of 108 sample groups: 4 types (control, 5%, 10%, and 15%) × 3 materials (PCL, PCL + Neem oil, PCL + Neem oil + Lavender oil) × 3 test subjects (Escherichia coli, Bacillus subtilis, and Staphylococcus aureus) × 3 test conditions (24 h, 48 h and 72 h). We prepared three samples per group. That means we have used 324 sample images for the disk diffusion assay. Figure 3 shows a group of sample images captured during the disk diffusion assay. 

*E. coli* had the lowest zone of inhibition at 5.25 mm. For PCL + 10% neem oil, there was an increase in the zone of inhibition for *B. subtilis* to 10 mm and 5 mm for *E. coli*. Similarly, with PCL and 15% neem oil, the largest zone of inhibition was 15 mm for *B. subtilis* and 7 mm for *E. coli*. The inhibition data were taken over three days, and the changes in inhibition areas are presented in Figure 2. In a previous study, the reported zones of inhibition for *E. coli*, *S. aureus*, and *B. subtilis* were 14 mm, 20 mm, and 26 mm, respectively [27].

The zone of inhibition for 15% PCL + lavender oil was 22 mm for *B. subtilis,* 17 mm for *S.aureus,* and 7 mm for *E. coli*. A similar study also reported that antimicrobial activity and inhibition growth were 14.65 mm for *E. coli* and 11.06 mm for *S. aureus* [28]. Finally, PCL, lavender, and the neem nanoemulsion mixture were tested for their antimicrobial properties. Our results indicate that the zone of inhibition for 15% mixed nanoemulsion is 24 nm for *B. subtilis* and 7.5 mm for *E. coli*. In a similar study, the antimicrobial activity of different plant essential oils was published, and the inhibitory value was 8 mm for *E. coli*. Our results are almost similar to other previous studies [26]. According to this study, one or two drops of lavender oil nanoemulsions increase their antibacterial activity. Lavender oil is typically employed as a flavoring agent, and it also assists in masking the neem odor.

### 3.5. Bactericidal Effect of Nanoemulsion

We tested the bactericidal effect of our 5%, 10%, and 15% nanoemulsions on *Escherichia coli*, *Bacillus subtilis*, and *Staphylococcus aureus*; Table 4 shows our results. This assay is used to evaluate an emulsion’s antibacterial properties. The advantage over the disk diffusion approach is that they are not dependent on the media’s or tested material’s diffusion characteristics [29]. 

Our results indicate that 5% neem oil nanoemulsion did not have any antimicrobial activity, as there was growth in all the tubes with *E. coli, S. aureus*, and *B.subtilis*. However, 10% neem oil nanoemulsion showed antimicrobial activity against *S. aureus* and *B. subtilis* only and not on *E. coli*. Nanoemulsions of 5% lavender oil did not inhibit the growth of *E. coli* but showed antimicrobial activity on the other two cultures. However, mixed neem and lavender nanoemulsions showed a synergistic antimicrobial effect on all three cultures tested, as there was no growth at 5%, 10%, or 15% concentration. In this study, the MIC values for neem oil and lavender oil were 15% for *E. coli*, 10% for *S. aureus* and *B. subtilis*, 10% for *E. coli,* and 5% for *S. aureus* and *B. subtilis*, respectively (Table 4). A previous study also reported that MIC values for lavender oil were 12.5% for *E. coli*, 6.25% for S*. aureus*, and 6.25% for *B. subtilis*, which is almost similar to this study result [30]. Our results in *E. coli*, *S. aureus*, and *B. subtilis* with neem and lavender oil nanoemulsions show their effectiveness as antimicrobials. This helps with recommending dosages, improving formulations, and making drugs that are safer for the environment [31]. We confirmed the bactericidal effect of all our nanoemulsions by performing a minimum inhibitory concentration (MIC) and minimum bactericidal concentration (MBC) assay. We performed a MBC by streaking a loopful of the test tube content from a bactericidal assay onto a nutrient agar plate with proper controls. The results are summarized in Table 5, and they confirm the results in Table 4. 

## 4. Conclusions

In summary, this research demonstrates the promising potential of using nanoemulsions containing neem and lavender oils in combination with polycaprolactone (PCL) as efficient and eco-friendly antimicrobial agents for air purification and disinfection. This study successfully established the enhanced antimicrobial efficacy of these nanoemulsions against common airborne pathogens, including *Escherichia coli*, *Bacillus subtilis*, and *Staphylococcus aureus*. Through rigorous testing, we optimized the physicochemical properties, minimum inhibitory concentration (MIC), and minimum bactericidal concentration (MBC) of these nanoemulsions. The droplet size ranges from neem oil are 23.92–119.15 nm, 21.86–115.15 nm for lavender oil, and 25.97–50.22 nm for their mixture. The PDI index (%) value range for neem oil is 100.45–140.44, 71.69–123.35 for lavender oil, and 95.25–131.78 for mixed oil. This study also found that the range of pH and viscosity for nanoemulsions at different concentrations was 5.8 to 6.6 pH and 0.372 to 2.101 cP. This research underscores the viability of integrating nanotechnology with natural essential oils to create innovative solutions for combating airborne bacterial contamination, thereby contributing to public health safety.

## Figures and Tables

**Table 1:** Viscosity, pH, and the formulation of nanoemulsions with different concentrations.

Samples	pH	Viscosity (cP)	Water Absorption (%)
5% neem oil	5.9 ± 0.09	2.101 ± 0.03	14.14 ± 0.63
10% neem oil	6.3 ± 0.11	1.807 ± 0.02	18.65 ± 0.99
15% neem oil	6.5 ± 0.12	1.440 ± 0.01	20.00 ± 0.55
5% lavender oil	5.8 ± 0.22	2.047 ± 0.05	23.15 ± 1.22
10% lavender oil	6.2 ± 0.05	1.050 ± 0.03	28.07 ± 1.33
15% lavender oil	6.3 ± 0.12	0.548 ± 0.02	31.45 ± 1.03
5% neem with lavender oil	6.2 ± 0.20	1.840 ± 0.03	16.36 ± 0.88
10% neem with lavender oil	6.5 ± 0.03	0.726 ± 0.01	21.53 ± 0.62
15% neem with lavender oil	6.6 ± 0.05	0.372 ± 0.01	26.34 ± 1.32

**Table 2:** Characteristics and thermodynamic stability of nanoemulsions at 4, 25, and 37 °C (value (nm) ± SD).

Formulation	Droplet Size nm (4 °C)	Droplet Size nm (25 °C)	Droplet Size nm (37 °C)	% PDI (4 °C)	% PDI (25 °C)	% PDI (37 °C)
Control (5% T)	81.47 ± 0.99	97.95 ± 0.63	105.52 ± 1.01	16.04 ± 0.13	16.09 ± 2.26	16.38 ± 1.22
Control (10% T)	12.09 ± 0.06	15.01 ± 0.03	24.72 ± 0.23	11.69 ± 0.99	12.45 ± 1.10	12.20 ± 1.09
Control (15% T)	9.04 ± 0.03	11.21 ± 0.02	9.83 ± 0.03	17.70 ± 1.01	18.02 ± 2.55	18.99 ± 1.26
Neem (5%)	119.15 ± 1.08	132.57 ± 1.13	148.17 ± 1.11	11.73 ± 1.09	13.37 ± 1.02	14.81 ± 1.44
Neem (10%)	28.84 ± 0.55	38.47 ± 0.92	46.99 ± 0.22	10.23 ± 0.88	11.01 ± 1.19	11.24 ± 1.05
Neem (15%)	23.92 ± 0.23	43.61 ± 0.55	58.87 ± 0.55	10.04 ± 0.69	10.22 ± 0.98	10.73 ± 0.92
Lavender (5%)	115.15 ± 0.66	121.48 ± 1.06	135.75 ± 1.03	7.16 ± 0.55	8.10 ± 0.82	8.78 ± 0.92
Lavender (10%)	29.16 ± 0.22	36.12 ± 0.52	39.2 ± 0.22	10.27 ± 1.06	11.54 ± 1.10	12.33 ± 1.10
Lavender (15%)	21.86 ± 0.09	52.78 ± 0.11	48.73 ± 0.11	7.47 ± 0.88	10.58 ± 1.22	11.10 ± 1.35
Neem + Lavender (5%)	50.22 ± 0.08	48.78 ± 0.44	57.59 ± 0.10	9.52 ± 1.02	11.05 ± 1.31	9.98 ± 0.99
Neem + Lavender (10%)	28.1 ± 0.33	41.9 ± 0.22	49.94 ± 0.15	12.11 ± 1.20	12.27 ± 1.01	13.17 ± 1.52
Neem + Lavender (15%)	25.97 ± 0.69	36.0 ± 0.36	41.01 ± 0.12	15.63 ± 0.35	6.80 ± 0.60	7.98 ± 0.97

Legend: T = Tween 80, nm = nanometer.

**Table 3:** The optical properties of nanoemulsions with different concentrations (mean ± SD).

Samples	L*	a*	b*	Δ*E*	*C*	*H*
5% control	42.91 ± 0.22 ^b^	−7.58 ± 0.05 ^b^	17.15 ± 0.03 ^c^	46.82 ± 0.25 ^c^	18.75 ± 0.05 ^a^	113.8 ± 0.77 ^b^
10% control	42.36 ± 0.11 ^d^	−5.89 ± 0.06 ^a^	15.24 ± 0.02 ^b^	45.40 ± 0.19 ^a^	16.33 ± 0.03 ^d^	111.1 ± 0.55 ^a^
15% control	44.14 ± 0.20 ^b^	8.12 ± 0.09 ^d^	9.99 ± 0.01 ^a^	45.97 ± 0.34 ^b^	12.87 ± 0.02 ^c^	50.8 ± 0.60 ^c^
5% neem oil	79.67 ± 0.09 ^c^	−0.22 ± 0.01 ^c^	7.70 ± 0.03 ^d^	80.0 ± 0.55 ^d^	7.70 ± 0.01 ^b^	91.6 ± 0.50 ^d^
10% neem oil	78.22 ± 0.10 ^a^	−0.64 ± 0.01 ^a^	7.33 ± 0.01 ^a^	78.56 ± 0.33 ^ab^	7.35 ± 0.01 ^b^	94.9 ± 0.50 ^bc^
15% neem oil	79.60 ± 0.23 ^bc^	−2.83 ± 0.02 ^b^	10.55 ± 0.01 ^bc^	80.34 ± 0.22 ^c^	10.92 ± 0.03 ^c^	105.0 ± 0.44 ^b^
5% lavender oil	80.57 ± 0.15 ^d^	−0.71 ± 0.01 ^c^	1.44 ± 0.01 ^d^	80.58 ± 0.45 ^b^	1.60 ± 0.01 ^ac^	116.2 ± 0.41 ^d^
10% lavender oil	82.32 ± 0.11 ^a^	−1.06 ± 0.02 ^a^	2.00 ± 0.02 ^c^	82.35 ± 0.41 ^d^	2.26 ± 0.01 ^d^	117.9 ± 0.60 ^a^
15% lavender oil	74.39 ± 0.15 ^b^	−2.15 ± 0.03 ^d^	2.64 ± 0.01 ^a^	74.46 ± 0.44 ^c^	3.40 ± 0.02 ^cd^	129.1 ± 0.50 ^a^
5% neem + lavender oil	77.97 ± 0.23 ^d^	0.37 ± 0.01 ^bc^	3.31 ± 0.01 ^c^	78.04 ± 0.35 ^a^	3.33 ± 0.01 ^b^	83.6 ± 0.66 ^c^
10% neem + lavender oil	81.13 ± 0.19 ^a^	−2.47 ± 0.01 ^c^	6.47 ± 0.05 ^b^	81.42 ± 0.55 ^b^	6.92 ± 0.01 ^d^	110.8 ± 0.51 ^b^
15% neem + lavender oil	78.63 ± 0.15 ^c^	−4.20 ± 0.01 ^a^	12.22 ± 0.03 ^d^	79.68 ± 0.35 ^cd^	12.92 ± 0.03 ^a^	108.9 ± 0.55 ^d^

Note: All values are means and standard errors of three replicates. The means in each column that are denoted by a distinct superscript letter differ significantly (*p* < 0.05). In the above equation, huge angle, H = tan^−1^ (b*/a*), and chroma, $C=\sqrt{{a^{*}}^{2}+{b^{*}}^{2}}$.

**Table 4:** Minimum inhibitory concentration (MIC) for different concentrations of nanoemulsions after 24 h.

Samples	*E. coli*	*S. aureus*	*B. subtilis*
5% neem oil	+++	+++	+++
10% neem oil	+++	---	---
15% neem oil	---	---	---
5% lavender oil	+++	---	---
10% lavender oil	---	---	---
15% lavender oil	---	---	---
5% neem + lavender oil	---	---	---
10% neem + lavender oil	---	---	---
15% neem + lavender oil	---	---	---

Each + or - symbol represents, respectively, the presence or absence of the microorganisms in one of the triplicate tubes.

**Table 5:** Minimum bactericidal concentration (MBC) for different concentrations of nanoemulsions after 24 h.

Samples	*E. coli*	*S. aureus*	*B. subtilis*
5% neem oil	+++	---	---
10% neem oil	+++	---	---
15% neem oil	---	---	---
5% lavender oil	+++	---	---
10% lavender oil	---	---	---
15% lavender oil	---	---	---
5% neem + lavender oil	---	---	---
10% neem + lavender oil	---	---	---
15% neem + lavender oil	---	---	---

Each + or - symbol represents, respectively, the presence or absence of the microorganisms in one of the triplicate plates.

## Data Availability

Data are contained within the article.
